# Epidemiological assessment of the severity of dengue epidemics in French Guiana

**DOI:** 10.1371/journal.pone.0172267

**Published:** 2017-02-14

**Authors:** Claude Flamand, Camille Fritzell, Christelle Prince, Philippe Abboud, Vanessa Ardillon, Luisiane Carvalho, Magalie Demar, Rachida Boukhari, Martine Papaix-Puech, Narcisse Elenga, Dominique Rousset, Séverine Matheus, Mathieu Nacher, Philippe Quenel, Félix Djossou

**Affiliations:** 1 Institut Pasteur de la Guyane, Cayenne, French Guiana; 2 Centre Hospitalier Andrée Rosemon, Cayenne, French Guiana; 3 Santé Publique France, Cayenne, French Guiana; 4 Centre Hospitalier de l’Ouest Guyanais, Saint-Laurent du Maroni, French Guiana; 5 Centre Médico-Chirurgical de Kourou, French Guiana; 6 Inserm UMR 1085-IRSET Institut de Rechercheen Santé, Environnementet Travail, EHESP, Rennes, France; Duke-National University of Singapore Graduate Medical School, SINGAPORE

## Abstract

**Background:**

Dengue fever is the most important arboviral infection that affects humans, particularly in tropical and subtropical regions. Here, we provide the first comprehensive overview of the severity of dengue epidemics in French Guiana.

**Methodology/Principal findings:**

We monitored hospitalized cases between 2008 and 2013. Detailed clinical features and biological parameters were collected on a daily basis from all cases. Among the 1,356 cases, 216 (16%) were classified according to the WHO 2009 classification as dengue without warning signs (WS), 926 (68%) were classified as dengue with WS and 214 (16%) were classified as severe dengue. The severity rates were similar between the three major epidemics that occurred during the study period, whereas the hospitalization rate was highest in 2013. Fluid accumulation, aspartate aminotransferase (ASAT) counts>193 IU/L and platelet counts<75,000 cells/mm^3^ were associated with dengue severity.

**Conclusions/Significance:**

Our findings provide a recent epidemiological description of the severity of dengue epidemics in French Guiana. These results highlight the potential impacts and consequences of implementing the WHO 2009 classification on hospital activity. Future studies should include virological and immunological investigations of well-documented serum samples.

## Introduction

Dengue fever is a human acute febrile disease caused by any of the four dengue virus (DENV) serotypes, and it is transmitted by infected *Aedes* mosquitoes. Recent estimates indicate that dengue fever is the most important arthropod-borne human disease, with infections primarily occurring in tropical and subtropical regions; there are 390 million dengue infections per year worldwide, of which 96 million are clinically apparent [1–3].

Dengue infection can cause a wide range of clinical manifestations, from syndromes that are self-limited to severe [4]. After the incubation period, clinical manifestations can be divided into three phases: an initial febrile phase lasting three to seven days; a critical phase around defervescence, during which complications appear in a small proportion of patients; and a spontaneous recovery phase. A small proportion of infected persons develop a severe form of the disease, including dengue hemorrhagic fever (DHF) or dengue shock syndrome, although with early diagnosis and proper supportive care, the fatality rate can be less than 1% [1]. However, there is currently no accurate method for the early prediction of disease severity, and cases of non-severe dengue without WS can develop into severe dengue (SD) [5–9].

Many clinical trials and studies have reported factors associated with the development of DHF, including DENV serotype, platelet counts≤75,000/mm^3^ and hematocrit values of 50%, a rise of more than 22% from baseline hematocrit levels, high viral load, and intense activation of the immune system, as well as various host conditions, including extremes of age, dengue immune status, genetic race (e.g., Caucasians), AB blood group, nutritional status and coexisting conditions [1,5–9].

Since the reemergence of dengue in Latin America during the 1960s, there has been a steady increase in the number of reported and severe cases associated with co-circulation of the four serotypes, with the past decade having the highest number of recorded cases to date [10–12]. Since the first DHF cases were reported in 1992 in French Guiana—a French overseas territory of 250,000 inhabitants located in South America—dengue transmission has followed a seasonal pattern punctuated by the co-circulation of several serotypes and increasingly frequent epidemics [13–19]. However, despite the reinforcement of surveillance systems, no published studies have given a comprehensive description of the epidemiology of dengue feverin terms of clinical characteristics, severity and trends over time [20,21].

From 2008 to 2013, all hospitalized confirmed cases of dengue were documented using an active data collection process. Here, we investigated the clinical features and biological parameters of the 1,356 hospitalized patients evaluated during this five-year period, and we provide a comprehensive description of the severity of recent major dengue outbreaks in French Guiana.

## Materials and methods

### Study design and population

French Guiana, one of the five countries of the Amazonian shield and located between Brazil and Surinam, is composed of two main geographical regions: a central urbanized area and coastal strip along the Atlantic Ocean where a large portion of the population lives, and a more remote area along the Surinamese and Brazilian frontiers. However, regardless of their area of residence, all hospitalized dengue cases are sent to one of three hospital centers located in the coastal strip. A hospital monitoring system was established between October 2008 and December 2013 by the Infectious and Tropical Disease Unit of Cayenne Hospital. This monitoring system was part of a multisource dengue epidemiological surveillance system coordinated by the Epidemiology Unit of the French Public Health Agency (Cire AG) in collaboration with the Arbovirus National Reference Center (CNR) of Pasteur Institute of French Guiana and other public health partners [15].

### Data collection from hospital care units

All patients having suggestive clinical features associated with a biological confirmation of dengue infection who were admitted to one of the three public hospitals of French Guiana (Cayenne, Kourou and Saint-Laurent) were enrolled in this study. Biological confirmation was obtained through positive nonstructural protein 1 (NS1) antigen detection or positive anti-DENV immunoglobulin M (IgM) or immunoglobulin G (IgG) detection or viral isolation or RT-PCR [22].

Clinical features, including baseline conditions and co-morbidities, pulse rate, blood pressure, temperature, presence of any bleeding manifestations and presence of any fluid accumulation, were collected daily from the time of admission until the patients were discharged.

Biological parameters, including blood counts, serum aspartate aminotransferase (ASAT), alanine aminotransferase (ALAT), neutrophils, lymphocytes, serum total protein, prothrombin, natremia, troponin, albuminuria, creatinine, creatine phosphokinase, C-reactive protein and hematocrit levels were also collected.

### Dengue classification and definitions

Based on clinical and biological features, each patient enrolled in the study was classified according to the WHO 2009 classification, which was used retrospectively from 2008 until the end 2010, and thereafter prospectively until 2013. All patients were classified by a single clinician as one of the following: dengue fever without WS, dengue with WS and SD. The patients were classified as having WS if they had abdominal pain or tenderness, persistent vomiting, clinical fluid accumulation, mucosal bleeding, lethargy, hepatomegaly or increased hematocrit(between 10% and 20% of baseline hematocrit) concurrent with a rapid decrease in platelet count (below 50 G/L on day 5).

SD was characterized by severe plasma leakage, severe bleeding or severe organ involvement [1]. Severe plasma leakage was defined as the presence of one or more of the following criteria: i) hypotensive shock assessed by prolonged capillary refill time, cold and clammy extremities, feeble or absent peripheral pulse volume, severe tachycardia, narrowed pulse pressure or unrecordable blood pressure or hypotension based on age; ii) evidence of fluid accumulation (pleural effusions or ascites) or respiratory distress; or iii)increased hematocrit levels (hematocrit variation > 20%) concurrent with a rapid decrease in platelet count. Clinical fluid accumulation was defined and assessed by the presence of peripheral edema or serous effusions (pleural or ascitic) of slight to medium severity.

### Epidemiologic surveillance system

A clinical surveillance system involving a sentinel network of 30 voluntary general practitioners and 17 remote Health Centers was established and implemented in 2006 by Cire AG to estimate the weekly incidence of clinical cases seen in consultation. A clinical case of dengue fever was defined by the occurrence of fever (≥38°C) with no evidence of other infection and associated with one or more non-specific symptoms, including headache, myalgia, arthralgia or retro-orbital pain [15].

Epidemic periods of dengue fever were defined according to the Program for Surveillance, Alert and Response (PSAGE) that was elaborated in 2008 by a local vector-borne diseases committee composed of epidemiologists, biologists, clinicians, entomologists and specialists in charge of vector control [15].

Dengue serotypes were identified for a random sample of approximately 30% of the confirmed cases using a nested RT-PCR technique, described by Lanciotti *et al*. [22], performed by the National Reference Center based at the Pasteur Institute in French Guiana.

### Ethics statement

The anonymized data collection issued from medical records and was authorized by regulatory authorities (CNIL- N° TFN1490159N). Laboratory surveillance data collection was approved by the Advisory Committee on Information Processing in Material research in the Field of Health (N°07.148) and was authorized by regulatory authorities (CNIL-N°1213498).

### Data analysis

To calculate hospitalization rates and the severity of epidemics, the confirmed hospitalized cases and patients with SD were compared to the total number of clinical cases estimated from the clinical surveillance system. Univariate analysis was performed using Kruskal-Wallis and Chi-square tests for comparisons between quantitative and categorical variables, respectively. Univariate and multivariate logistic regression were used to study the adequacy of theWHO 2009 classification and to identify factors associated with dengue severity. Regarding dengue severity, the dependent variable used in the models was the dengue classification type (“SD” vs “Dengue with or without WS”). The level of statistical significance was set to P = 0.05. Statistical analysis was performed using the STATA12 software program (Stata Corp., College Station, TX, USA).

A value for the nominal variable “transaminases” was created using the maximum value between ASAT and ALAT levels, which was then divided into three categories: [0–32], [32–62] and [>62]. A quantitative variable “delta-hemat” was calculated using the equation $\frac{\text{maximimum hematocrit}-\text{hematocrit at convalescence}}{\text{hematocrit at convalescence}}*100$ to represent hematocrit variation. As hospitalized patients were systematically receiving antipyretics, it was difficult to determine the time of defervescence in all cases. We observed, however, that this generally occurred between days 3 and 7 of the illness, consistent with the WHO 2009 recommendations. Therefore, biological features on day 5 of the illness were used to assess infection evolution.

## Results

### Description of hospitalized cases

**Fig 1:**
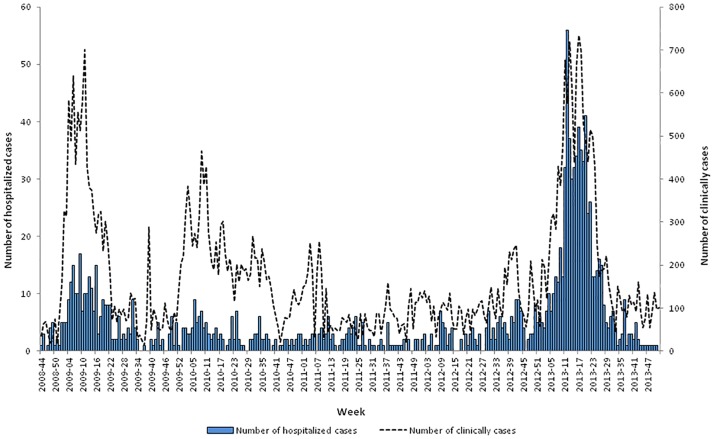
Weekly number of hospitalized dengue cases and the distribution of clinically estimated cases (n = 1356), French Guiana, 2008–2013.

**Fig 2:**
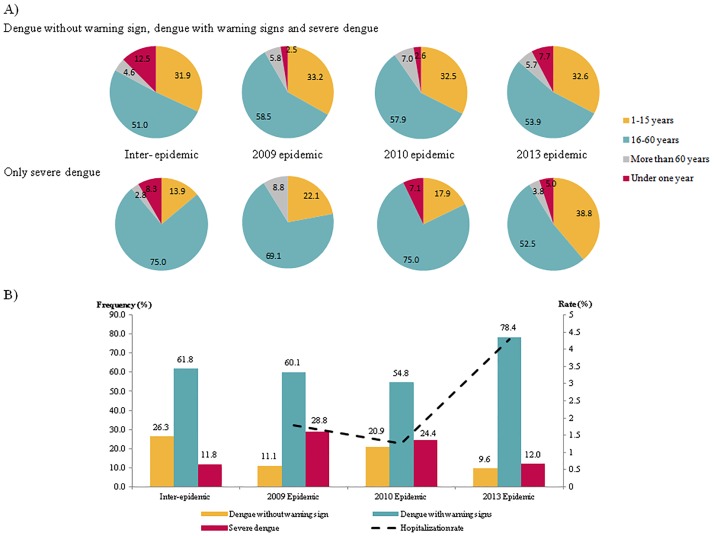
Distribution of epidemiological features according to the three outbreaks, (n = 1356), French Guiana, 2008–2013. A) Age distribution of individuals by epidemic. B) Distribution of categories based on 2009 WHO criteria and hospitalization rates.

Between October 2008 and December 2013, 1,356 hospitalized cases were included in the study, of which 243 were hospitalized during the 2009 epidemic, 115 in 2010 and 694 in 2013 (Fig 1). As shown in Table 1, the majority of cases were admitted at Cayenne hospital (65%), females were over-represented in the cohort (54%), and more than 20% of hospitalized cases were children under seven, whereas only 5% were more than 60 years old. Overall, 216 (16%) cases were classified as dengue without WS, 926 (68%) as dengue with WS and 214 (16%) as SD. NS1 tests, IgM and IgG ELISA procedures were performed to diagnose dengue infection, and 848, 374 and 189 patients tested positive for these three tests, respectively. The 1,356 patients ranged in age from birth to 90 years, with a mean of 25 years (±19.9 years). The analysis of sociodemographic features revealed that the age distribution differed significantly according toclinical category (P<0.001). Overall, hospitalized cases of patients under one year of age were under-represented (4%) among SD cases, whereas patients 16–60 years old were over-represented (64%) among severe cases (Fig 2A).

**Table 1:** Distribution of hospital centers and sociodemographic variables among hospitalized cases (n = 1356), French Guiana, 2008–2013.

Factors	Count (N)	Frequency (%)
**Hospital Center**		
*Cayenne*	885	65.27
*Sain-Laurent*	176	12.98
*Kourou*	295	21.75
**Gender**		
*Male*	628	46.31
*Female*	728	53.69
**Age**		
*<1*	100	7.4
*[1–6]*	203	15.0
*[7–15]*	240	17.7
*[16–44]*	581	42.8
*[45–60]*	147	10.8
*>60*	74	5.5
*ND*	11	0.8
**Total**	1356	100

### Comparisons over time

As shown in Fig 2A, the proportion of hospitalized cases under one year of age was higher during the 2013 epidemic (7.7%), whereas this proportion was similar in 2010 and 2013 (2.5 and 2.6%, respectively). Among SD cases, there were no hospitalized cases under one year of age in 2009; this proportion varied between 2010 and 2013 decreasing from 7 to 5%. There were more hospitalized cases under one year of age during inter-epidemic periods than during epidemic periods.

A comparison of the clinical category distributions revealed an increase in the hospitalization rate in 2013 (4.3% vs. 1.8% in 2009 and 1.3% in 2010) (Fig 2B). By contrast, the proportion of SD cases did not show the same trend, gradually but significantly decreasing from 28.8% in 2009 to 12% in 2013 (P<0.01). This reduction was concomitant with an increase in dengue cases with WS (60.1% in 2009 vs. 78.4% in 2013). The rise in the hospitalization rate during 2013 was not followed by an increased proportion of severe hospitalized dengue cases.

### Epidemic severity

Although the 2009 and 2013 epidemics differed in terms of magnitude, they were similar in terms of severity rates (0.5% and 0.6%), whereas the 2010 epidemic had a lower severity rate (0.3%) (Table 2). Overall, there was a statistically significant difference in terms of severity rates among the three epidemics (P = 0.006). We also noted that the hospitalization rate substantially increased between 2009 and 2013 (1.7 to 5.2%, P<10^−3^). The 2013 epidemic had the highest impact on hospital admissions during the study period, with 694 hospitalizations. However, the duration of hospitalization did not show any significant variation between epidemics (Table 2).

**Table 2:** Description of indicators related to epidemic severity in hospitalized patients, French Guiana, 2008–2013.

	Epidemic		
	2009	2010	2013
Dengue without WS	27 (11%)	24 (21%)	85 (12%)
Dengue with WS	146 (60%)	63 (55%)	529 (76%)
Severe dengue	70 (29%)	28 (24%)	80 (12%)
Total number of hospitalized cases	243	115	694
Estimated number of clinical cases	13900	9220	13240
Death	2	1	6
**Severity rate (%)**	**0.5**	**0.3**	**0.6**
**Hospitalization rate**	**1.7**	**1.2**	**5.2**
**Hospitalization duration**	**3.98**	**4**	**3.97**

### Adequacy of the application of WHO 2009 classification

Results from the logistic regression analyses indicated the adequacy of applying the WHO 2009 classification to the clinical signs reported among hospitalized patients in French Guiana. Patients were more likely to be classified as severe cases if they were older than 16 years or they showed the presence of petechia, epistaxis, fluid accumulation, faintness, prolonged hospitalization delay, a long hospitalization duration, prolonged symptom duration, a platelet count lower than 75,000 cells/mm^3^, transaminases level higher than 62 UI/L or a variation in hematocrit levels (Table 3). The multivariate analysis identified potential predictors of SD, which included the presence of fluid accumulation, transaminases level higher than 62 UI/L, an increase inhematocrit levels between consultations and an age greater than 16. Moreover, this analysis also identified total serum protein as a protective factor. The retrospective implementation of this classification system for cases admitted in 2009 and 2010 allowed us to make an epidemiologic comparison between the outbreaks during the study period.

**Table 3:** Crude and adjusted associations between possible risk factors and development of severe dengue disease using logistic regression.

Variable	Frequency (%)	Univariate Model			Multivariate Model		
	Yes/No	OR*	95% CI	p-value	OR**	95% CI	p-value
**Petechia**	4.90/95.1	2	[1.12–3.57]	0.019			
**Purpura**	0.40/99.6	1.24	[0.14–11.1]	0.846			
**Epistaxis**	4.50/95.5	2.95	[1.67–5.20]	<0.001			
**Dehydration**	5.50/97.5	0.93	[0.48–1.80]	0.833			
**Neuropsychiatric disorders**	2.0/98.0	1.98	[0.81–4.80]	0.130			
**Hepatomegaly**	1.70/98.3	1.9	[0.73–4.92]	0.183			
**Headache**	34.0/66.0	0.67	[0.48–0.93]	0.018			
**Fluid accumulation**	2.80/97.2	14.1	[6.65–29.7]	<0.001	9.14	[2.47–33.9]	0.001
**Faintness**	36.7/63.6	0.63	[0.46–0.87]	<0.01			
**Rash**	12.8/87.2	0.75	[0.47–1.21]	0.252			
**Nausea**	36.7/63.6	0.87	[0.63–1.18]	0.364			
**Abdominal pain**	25.3/74.7	1.28	[0.92–1.77]	0.144			
**Asthenia**	18.4/81.6	1.38	[0.83–2.27]	0.207			
**Hospital delay (day)**^#^	.	1.09	[1.02–1.17]	0.012			
**Hospital duration (day)**	.	1.2	[1.13–1.27]	<0.001			
**Symptoms-duration (day)**	.	1.08	[1.04–1.12]	<0.001			
**Age>16 vs ≤16 years**	59.9/40.1	1.6	[1.17–2.20]	0.003	2.05	[1.14–3.69]	0.016
**Lymphocytes (/mm3)**	.	1.06	[0.92–1.23]	0.387			
**Neutrophils (/mm**^**3**^**)**	19.4/80.6	1.04	[0.95–1.13]	0.329			
**Platelets ≤75000 vs>75000 (mm**^**3**^**)**	37.2/62.8	2.25	[1.60–3.18]	<0.001			
**Albuminuria (g/L)**	.	0.84	[0.74–0.96]	0.016			
**Natremia (mmol/L)**	.	0.99	[0.96–1.02]	0.710			
**Troponine (μg/L)**	.	0.4	[0.00–158]	0.766			
**Transaminases >62 vs ≤62 (UI/L)**	90.3/9.7	2.97	[1.49–5.94]	<0.01	3.62	[1.63–8.08]	0.002
**Creatinine (μmol/L)**	.	1	[0.99–1.01]	0.861			
**Cpk (UI/L)**	.	0.99	[0.99–1.00]	0.708			
**Crp (mg/L)**	.	1	[0.99–1.01]	0.297			
**Temperature (°C)**	.	1.03	[0.86–1.23]	0.768			
**Prothrombin (%)**	.	1.01	[0.99–1.02]	0.545			
**Serum total protein (g/L)**	.	0.96	[0.93–0.98]	<0.01	0.97	[0.94–0.99]	0.048
**Hematocrit (%)**	.	1	[0.97–1.03]	0.933			
**Delta_hematocrit**	.	2.65	[2.16–3.26]	<0.001	2.4	[1.66–3.46]	<0.001
**Immunosuppression**	.	0.79	[0.28–2.28]	0.671			
**Sex (M vs W)**	46.8/53.2	0.9	[0.68–1.20]	0.503			
**Pregnancy**	9.1/90.9.	0.77	[0.38–1.58]	0.482			
**Comorbidity**	22.5/77.5	0.81	[0.46–1.46]	0.498			

OR*, crude odds ratio; OR**, adjusted odds ratio; CI, confidence interval

^#^ Delay between the onset of the symptoms and the date of hospitalization

### Serotypes

During the study, laboratory results identified 438 dengue subtypes. The distribution of serotypes was significantly different between the epidemics (P<0.001). The 2009 epidemic was caused predominantly by DENV-1 (67.6%) and DENV-4 (27.0%). DENV-2 was isolated throughout 2010, although the predominance of DENV-1 and DENV-4 were 48.8% and 43.9%, respectively. In 2013, a clear predominance of DENV-2 was observed, representing 96.7% of hospitalized cases. The distributions of the four serotypes were significantly different betweenclinical categories (P = 0.049). The DENV-2 serotype was more strongly associated with dengue severity (75.3% among SD, 74.7% among dengue with WS and 55.2% among dengue without WS) than was DENV-1 (13.5% among SD, 14.1% among dengue with WS and 37.9% among dengue without WS). DENV-1 was therefore over-represented among dengue cases without WS, whereas DENV-2 was under-represented in this category.

### Evolution of biological parameters

**Fig 3:**
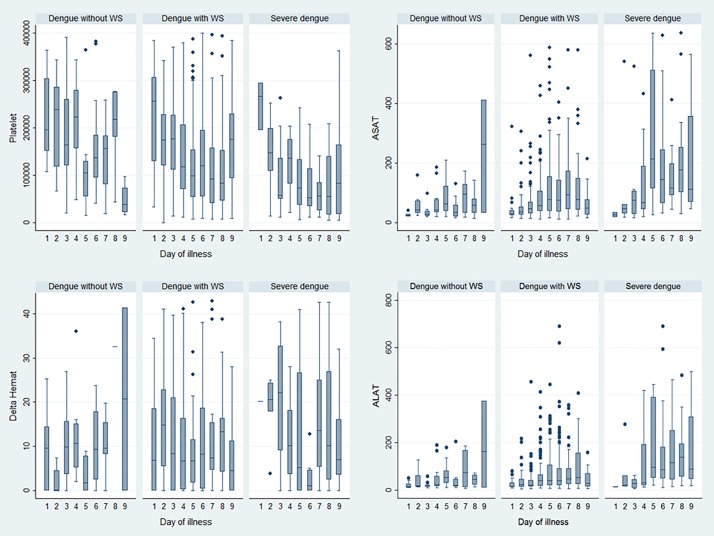
Box plots describing changes in ASAT, ALAT, platelet count and hematocrit variations during illness evolution among the three categories (dengue without WS, dengue with WS and severe dengue), French Guiana, 2008–2013.

Transaminase (ASAT and ALAT) levels progressively increased between days 1 and 5 days of the illness (Fig 3), with day 1 defined as the onset of fever. This trend was primarily observed in patients with WS and in SD. Platelet counts rapidly decreased during the critical phase between days 3 and day 2 in patients with WS and with SD, respectively. These decreases were concomitant with increased hematocrit levels.

## Discussion

This study was the first of its kind conducted in French Guiana describing the clinical and biological features associated with hospitalized dengue cases. The five-year system of monitoring hospitalized cases allowed us to describe the dynamics, magnitude and severity of the three epidemics that occurred since 2008. The impacts of the outbreaks on hospital activity were considerable in 2013, moderate in 2009 and minimal in 2010. The hospitalization rate was the highest during the 2013 outbreak (4.2%), which was noteworthy in terms of duration and number of estimated cases.

The retrospective implementation of the WHO 2009 classification for both epidemics among hospitalized cases enabled us to compare the severity of the epidemics. Although the number of admissions substantially increased in 2013, the severity rates were similar in 2009 and 2013. These results may be linked to increased awareness due to the WHO 2009 classification recommending that patients with any WS should be admitted for close observation [1]. This new classification has a high potential for facilitating case management and disease surveillance [23,24], and thus, could result fewer SD cases among hospitalized patients. Several studies assessing the usefulness of the revised dengue classification system have discussed its limitations, particularly the lack of specificity for some criteria, and they also noted a rise in the admission rate and increased workloads for medical professionals [23–26]. Furthermore, the impact of the classification application may have been reinforced in 2013 by the death of a young adult, which was highly covered by the media, at the beginning of the outbreak.

Health care policies recommend immediate admission for vulnerable patients (infants and elderly individuals) with dengue-like syndrome to avoid severe illness. Professional practices following these recommendations could have explained the small number of infants with severe forms of the disease (7%). In addition, many studies have noted that secondary infection with another serotype could be a risk hazard for SD [27–30]. This hypothesis could explain the underrepresentation of infants among severe cases and the overrepresentation of adults in this category.

According to the univariate analysis, we found that the presence of petechia, epistaxis, clinical fluid accumulation, hepatomegaly, low pulse rate and abdominal pains were associated with SD. At day 5 of the illness, low albuminuria, natremia and serum total protein levels as well as low platelet counts were associated with SD. Patients with SD had higher ASAT and ALAT levels and higher hematocrit variations at day 5 than patients classified with dengue without WS or with WS. We found that low platelet count and transaminases levels were significantly associated with SD. Recent studies regarding the 2009 WHO classification confirmed these results [31,32]. In addition to these factors, serum total protein, natremia and albuminuria levels appeared to be protective factors, although the crude odds ratios (OR) were only slightly protective. Regarding serum total protein levels, this result was not surprising given that a rise in hematocrit is concomitant with a drop in serum total protein levels.

One limitation of this study was that inclusion was restricted to hospitalized cases, which does not allow us to generalize our findings to all dengue cases. Indeed, our results regarding the factors associated with SD could be different if ambulatory cases were included in the study.

One of the strengths of our study was that all hospitalized cases between November 2008 and December 2013 admitted at any of the three hospitals for the whole territory were enrolled ina large study population (n = 1,356), giving our results good power and representation. Additionally, a single clinician classified all of the cases, leading to high quality data and avoiding biases related to variations in medical practices. In addition, the daily data collection from the time of admission until discharge allowed us to describe the clinical evolution and to study the clinical and biological features throughout the hospitalization period. We cannot rule out the possibility that clinical signs and symptoms used in the WHO 2009 system may not have been stringently collected prior to its implementation. Nevertheless, clinical features collected during the study period appeared consistent with the proper implementation of WHO 2009 classification.

The pathophysiology of SD appears to be multifactorial, entailing complex interactions among viral factors, host genetics and the immunologic background of the host, the most important being prior to exposure to dengue virus. Hyperendemicity with different serotypes is believed to be one of the most significant factors influencing dengue severity [33]. Future studies should assess the immune status of the patients and should evaluate the role of secondary infections in likelihood of severe disease development.

## Conclusion

We presented a comprehensive epidemiological description of the severity of dengue epidemics in French Guiana. We also identified potential new clinical and biological predictive factors of dengue severity, although further work in identifying other virological and immunological markers is needed to improve our knowledge of the risk factors in French Guiana.

## References

[1] World Health Organization (WHO). Dengue guidelines for diagnosis, treatment, prevention and control. http://www.who.int/rpc/guidelines/9789241547871/en/. Accessed 4 September 2014. 23762963

[2] Brady OJ, Gething PW, Bhatt S, Messina JP, Brownstein JS, Hoen AG, et al Refining the global spatial limits of dengue virus transmission by evidence-based consensus. PLoS Negl Trop Dis. 2012;6: e1760 10.1371/journal.pntd.0001760 22880140 PMC3413714

[3] Bhatt S, Gething PW, Brady OJ, Messina JP, Farlow AW, Moyes CL, et al The global distribution and burden of dengue. Nature. 2013;496: 504–507. 10.1038/nature12060 23563266 PMC3651993

[4] Rigau-Pérez JG, Clark GG, Gubler DJ, Reiter P, Sanders EJ, Vance Vorndam A. Dengue and dengue haemorrhagic fever. Lancet. 1998;352: 977. 10.1016/s0140-6736(97)12483-7 9752834

[5] Srikiatkhachorn A, Green S. Markers of dengue disease severity. Curr Top Microbiol Immunol. 2010;338: 67–82. 10.1007/978-3-642-02215-9_6 19802579

[6] Khan MI, Anwar E, Agha A, Hassanien NS, Ullah E, Syed IA, et al Factors predicting severe dengue in patients with dengue fever. Mediterr J Hematol Infect Dis. 2013;5: e2013014 23505602 10.4084/MJHID.2013.014 PMC3591277

[7] Pawitan JA. Dengue virus infection: predictors for severe dengue. Acta Med Indones. 2011;43: 129–135. 21785176

[8] Lam PK, Tam DT, Diet TV, Tam CT, Tien NT, Kieu NT, et al Clinical characteristics of dengue shock syndrome in Vietnamese children: a 10-year prospective study in a single hospital. Clin Infect Dis. 2013;57: 1577–1586. 10.1093/cid/cit594 24046311 PMC3814826

[9] Marón GM, Escobar GA, Hidalgo EM, Clara AW, Minniear TD, Martinez E, et al Characterization of dengue shock syndrome in pediatric patients in El Salvador. Pediatr Infect Dis J. 2011;30: 449–450. 10.1097/INF.0b013e318212ab8e 21490492

[10] Pinheiro FP, Corber SJ. Global situation of dengue and dengue haemorrhagic fever, and its emergence in the Americas. World Health Stat Q. 1997;50: 161–169. 9477544

[11] Pan American Health Organization. Surveillance–featured surveillance items. http://www.paho.org/hq/index.php?option=com_topics&view=article&id=1&Itemid=40734. Accessed 19 January 2015.

[12] Brathwaite DO, San Martín JL, Montoya RH, del Diego J, Zambrano B, Dayan GH. The history of dengue outbreaks in the Americas. Am J Trop Med Hyg. 2012;87: 584–593. 10.4269/ajtmh.2012.11-0770 23042846 PMC3516305

[13] Quénel P, Rosine J, Cassadou S, Ardillon V, Blateau A, Matheus S, et al Epidémiologie de la dengue dans les Départements francais d’Amériques. Bull Epidemiol Hebd. 2011;33–34: 358–363.

[14] L'Azou M, Taurel AF, Flamand C, Quénel P. Recent epidemiological trends of dengue in the French territories of the Americas (2000–2012): a systematic literature review. PLoS Negl Trop Dis. 2014;8: e3235 10.1371/journal.pntd.0003235 25375627 PMC4222734

[15] Flamand C, Quenel P, Ardillon V, Carvalho L, Bringay S, Teisseire M. The epidemiologic surveillance of dengue-fever in French Guiana: when achievements trigger higher goals. Stud Health Technol Inform. 2011;169: 629–633. 21893824

[16] Adde A, Roucou P, Mangeas M, Ardillon V, Desenclos JC, Rousset D, et al Predicting dengue fever outbreaks in French Guiana using climate indicators. PLoS Negl Trop Dis. 2016;10: e0004681 10.1371/journal.pntd.0004681 27128312 PMC4851397

[17] Carles G, Peiffer H, Talarmin A. Effects of dengue fever during pregnancy in French Guiana. Clin Infect Dis. 1999;28: 637–640. 10.1086/515144 10194092

[18] Hanf M, Friedman E, Basurko C, Roger A, Bruncher P, Dussart P, et al Dengue epidemics and adverse obstetrical outcomes in French Guiana: a semi-ecological study. Trop Med Int Health. 2014;19: 153–158. 10.1111/tmi.12240 24341915 PMC5104834

[19] Flamand C, Fabregue M, Bringay S, Ardillon V, Quénel P, Desenclos JC, et al Mining local climate data to assess spatiotemporal dengue fever epidemic patterns in French Guiana. J Am Med Inform Assoc. 2014;21: e232–e240. 10.1136/amiajnl-2013-002348 24549761 PMC4173173

[20] Djossou F, Vesin G, Bidaud B, Mosnier E, Simonnet C, Matheus S, et al Incidence and predictive factors of central nervous system dysfunction in patients consulting for dengue fever in Cayenne Hospital, French Guiana. PLoS One. 2016;11: e0150828 10.1371/journal.pone.0150828 26981859 PMC4794179

[21] Djossou F, Vesin G, Walter G, Epelboin L, Mosnier E, Bidaud B, et al Incidence and predictive factors of transaminase elevation in patients consulting for dengue fever in Cayenne Hospital, French Guiana. Trans R Soc Trop Med Hyg. 2016;110: 134–140. 10.1093/trstmh/trv117 26822606

[22] Lanciotti RS, Calisher CH, Gubler DJ, Chang GJ, Vorndam AV. Rapid detection and typing of dengue viruses from clinical samples by using reverse transcriptase-polymerase chain reaction. J Clin Microbiol. 1992;30: 545–551. 1372617 10.1128/jcm.30.3.545-551.1992 PMC265106

[23] Barniol J, Gaczkowski R, Barbato EV, da Cunha RV, Salgado D, Martínez E, et al Usefulness and applicability of the revised dengue case classification by disease: multi-centre study in 18 countries. BMC Infect Dis. 2011;11: 106 10.1186/1471-2334-11-106 21510901 PMC3098176

[24] Deen JL, Harris E, Wills B, Balmaseda A, Hammond SN, Rocha C, et al The WHO dengue classification and case definitions: time for a reassessment. Lancet. 2006;368: 170–173. 10.1016/S0140-6736(06)69006-5 16829301

[25] Alexander N, Balmaseda A, Coelho IC, Dimaano E, Hien TT, Hung NT, et al Multicentre prospective study on dengue classification in four South-East Asian and three Latin American countries. Trop Med Int Health. 2011;16: 936–948. 10.1111/j.1365-3156.2011.02793.x 21624014

[26] Leo YS, Gan VC, Ng EL, Hao Y, Ng LC, Pok KY, et al Utility of warning signs in guiding admission and predicting severe disease in adult dengue. BMC Infect Dis. 2013;13: 498 10.1186/1471-2334-13-498 24152678 PMC4015176

[27] Wichmann O, Hongsiriwon S, Bowonwatanuwong C, Chotivanich K, Sukthana Y, Pukrittayakamee S. Risk factors and clinical features associated with severe dengue infection in adults and children during the 2001 epidemic in Chonburi, Thailand. Trop Med Int Health. 2004;9: 1022–1029. 10.1111/j.1365-3156.2004.01295.x 15361117

[28] Wichmann O, Gascon J, Schunk M, Puente S, Siikamaki H, Gjørup I, et al Severe dengue virus infection in travelers: risk factors and laboratory indicators. J Infect Dis. 2007;195: 1089–1096. 10.1086/512680 17357044

[29] Monath TP. Dengue: the risk to developed and developing countries. Proc Natl Acad Sci U S A. 1994;91: 2395–2400. 8146129 10.1073/pnas.91.7.2395 PMC43378

[30] Vaughn DW. Invited commentary: dengue lessons from Cuba. Am J Epidemiol. 2000;152: 800–803. 11085390 10.1093/aje/152.9.800

[31] Lima FR, Croda MG, Muniz DA, Gomes IT, Soares KR, Cardoso MR, et al Evaluation of the traditional and revised world health organization classifications of dengue cases in Brazil. Clinics (Sao Paulo). 2013;68: 1299–1304. 24212835 10.6061/clinics/2013(10)02 PMC3798712

[32] Jayaratne SD, Atukorale V, Gomes L, Chang T, Wijesinghe T, Fernando S, et al Evaluation of the WHO revised criteria for classification of clinical disease severity in acute adult dengue infection. BMC Res Notes. 2012;5: 645 10.1186/1756-0500-5-645 23167997 PMC3534490

[33] Thomas L, Verlaeten O, Cabié A, Kaidomar S, Moravie V, Martial J, et al Influence of the dengue serotype, previous dengue infection, and plasma viral load on clinical presentation and outcome during a dengue-2 and dengue-4 co-epidemic. Am J Trop Med Hyg. 2008;78: 990–998. 18541782

